# Transcriptomic analysis reveals distinct adaptive molecular mechanism in the hippocampal CA3 from rats susceptible or not-susceptible to hyperthermia-induced seizures

**DOI:** 10.1038/s41598-023-37535-w

**Published:** 2023-06-24

**Authors:** Silvia Y. Bando, Fernanda B. Bertonha, Pedro H. N. Menezes, André K. Takahara, Nathália A. Khaled, Paula Santos, Mara S. Junqueira, Roberto M. Cesar, Carlos A. Moreira-Filho

**Affiliations:** 1 Department of Pediatrics, Faculdade de Medicina da Universidade de São Paulo, São Paulo, SP 05403-900 Brazil; 2 Department of Radiology and Oncology, Centro de Investigação Translacional em Oncologia–Instituto do Câncer do Estado de São Paulo, Faculdade de Medicina da Universidade de São Paulo, São Paulo, SP 05403-000 Brazil; 3 Department of Computer Science, Instituto de Matemática e Estatística da Universidade de São Paulo, São Paulo, SP 05508-040 Brazil

**Keywords:** Neurological disorders, Epilepsy

## Abstract

Febrile seizures during early childhood are a relevant risk factor for the development of mesial temporal lobe epilepsy. Nevertheless, the molecular mechanism induced by febrile seizures that render the brain susceptible or not-susceptible to epileptogenesis remain poorly understood. Because the temporal investigation of such mechanisms in human patients is impossible, rat models of hyperthermia-induced febrile seizures have been used for that purpose. Here we conducted a temporal analysis of the transcriptomic and microRNA changes in the ventral CA3 of rats that develop (HS group) or not-develop (HNS group) seizures after hyperthermic insult on the eleventh postnatal day. The selected time intervals corresponded to acute, latent, and chronic phases of the disease. We found that the transcriptional differences between the HS and the HNS groups are related to inflammatory pathways, immune response, neurogenesis, and dendritogenesis in the latent and chronic phases. Additionally, the HNS group expressed a greater number of miRNAs (some abundantly expressed) as compared to the HS group. These results indicate that HNS rats were able to modulate their inflammatory response after insult, thus presenting better tissue repair and re-adaptation. Potential therapeutic targets, including genes, miRNAs and signaling pathways involved in epileptogenesis were identified.

## Introduction

Mesial temporal lobe epilepsy (MTLE) is the most common partial epilepsy in adults, with hippocampal sclerosis as the main pathological alteration^1^. Febrile insult in early childhood is the most common precipitating cause of MTLE^2^. About 13% of people with epilepsy have a history of febrile seizures^3,4^, with the febrile insult accounting for about 25% of cases of epileptic seizures in early childhood^5^. Additionally, about 7% of the infants and young children undergoing febrile seizures will develop epilepsy^2,6^.

MTLE and hippocampal sclerosis associated with febrile insult has been studied both in human patients and in animal models. Several studies revealed that hippocampal transcriptomic alterations occur simultaneously with insults and crises^2,7–10^. However, all the studies in MTLE patients were performed in hippocampal explants obtained during epilepsy surgery, making impossible to perform a temporal analysis of the transcriptomic changes during epileptogenesis, or across different phases of the disease. For this reason, research using animal models is imperative.

The use of rats for the study of prolonged hyperthermic-induced epilepsy is well established and mimics the febrile seizure in human infants^11–14^. Hyperthermic insults promote hyperexcitatory changes in the limbic system of rats triggering seizures and histological and electrophysiological alterations^15^. This model reproduces several characteristics similar to epilepsy in humans, such as early seizures, behavioral changes and myoclonus^16^, as well as synaptic and histological reorganizations in granule cells of the dentate gyrus (DG)^17^. Moreover, in a pilocarpine model, the CA3 region showed increased epileptogenesis when compared to the DG, covering most of the alterations that occurred in the transcript and protein levels^18^.

In addition, CA3 neuronal networks play a crucial role in memory information processes, with the ventral CA3 circuitry being the preferred area where post-ischemic hyperexcitability occurs^19^. Also, temporal analysis in the ventral CA3 showed several transcriptomic changes related to inflammation, neuronal differentiation and synaptic transmission^14^.

However, some relevant gaps need to be filled, especially regarding the study of changes in the molecular mechanism of the hippocampus that make rats exposed to hyperthermia prone to develop or not chronic seizures. Studies in this line of research may help to clarify the molecular mechanisms related to the hippocampus re-adaptation after the hyperthermic insult, subsequently rendering the brain susceptible or not to seizure development. Moreover, the identification of such molecular pathways may pave the way for the discover of potential therapeutic targets that could serve to interrupt epileptogenesis onset or MTLE progression.

Here, we investigated temporal transcriptomic and miRNA expression changes in the ventral CA3 of Wistar rats, since hyperexcitability of the hippocampus preferentially occurs in this region of the brain^19^, which is homologous to the human anterior hippocampus and related to histological changes in patients with MTLE^20^. The pups were submitted to hyperthermic insult on the 11th postnatal day. The four time-intervals analyzed corresponded to acute, latent, and chronic phases of the disease^12–14^.

## Materials and methods

### Animal model

The Ethics Committee on the Use of Animal (CEUA) of Faculdade de Medicina, University of São Paulo approved this study under the number 460/13. All experimental procedures followed the National Council for the Control of Animal Experimentation (CONCEA), in accordance to the ARRIVE guidelines. All efforts were made to minimize animal suffering and the number of animals used. Rats were maintained under standard environmental conditions (a temperature of 22 ± 2 °C, 12 h light-12 h dark cycle, and 55% ± 3% humidity) in propylene cages (50 × 34 × 27 cm, two or three rats per cage) with ad libitum access to food and water.

On the eleventh postnatal day (P11), all animals—except for those from the control group—were placed into a glass box with incandescent lamps (40W) and submitted to hyperthermia (39.5 to 42.3 °C) until reaching the core body temperature of 39 °C for 45 min and then monitored under euthermic conditions for 1 h. Control group animals were also placed in the glass box but not exposed to the incandescent lamps. Rectal temperatures were measured at baseline and recovery time, and every 10 min during the hyperthermic induction. Animals exposed to hyperthermic conditions reached 39.5 ± 0.5 °C in a similar time: the HS group in 27 ± 6 min and the HNS group in 25 ± 5 min. It is interesting to note that baseline and recovery core temperatures in the HNS group were higher compared to the HS group (p < 0.001). The HNS and HS groups presented respectively 34.6 ± 0.8 °C and 33.7 ± 0.8 °C at baseline, and 35.4 ± 1.6 °C and 33.2 ± 0.6 °C at recovery time. The control group core body temperature was maintained at 33.5 ± 0.5 °C throughout the experimental period. Behavioral hyperthermia-induced seizures in the post-induction period were classified according to Racine scale: orofacial automatisms (stage 1); head nodding (stage 2); forelimb clonus (stage 3); forelimb clonus with rearing (stage 4); and forelimb clonus with rearing and fallings (stage 5).

### Experimental design and time intervals

Only male rats were used in this study to minimize influences due to sexual dimorphism^21^. Sixty-two male rats were selected. The animals exposed to hyperthermic condition were divided into two groups: (i) HS (seizure, n = 22) group was comprised by animals presenting behavioral seizures classified as Racine’s stage 2 or higher; (ii) HNS group (n = 21) included animals that did not present seizures after the hyperthermic insult. The control (CT, n = 19) group was comprised by animals kept in euthermic conditions. The time intervals were based on previous studies that related time intervals and seizure resistance or susceptibility after pharmacological induction and recurrent seizures. In fact, a decrease in behavioral seizures induced by pentylenetetrazol was observed after 24 h (P12) and 20 days (P30) after hyperthermia induction^21,22^. Animals submitted to hyperthermia are prone to seizures after 50 days (P60) and P90 when treated with a sub-convulsive kainate^23,24^. Also, in this experimental model, 90% of the animals show epileptiform discharges in some degree^12^ and 45% exhibited spontaneous recurrent seizures around 3 to 4 months (P120) of age^13^.

### Ventral CA3 microdissection and RNA extraction

Ventral CA3 samples were collected at the selected time intervals for transcriptomic evaluation of the acute (P12), latent (P30 and P60) and chronic (P120) stages of the experimental model. The microdissection was performed as previously described by Gorter et al.^25^. After decapitation, temporal lobe and hippocampus were removed by incision at the ventrocaudal part under the rhinal fissure 5 mm posterior to bregma. Then, ventral CA3 region was collected and placed in Eppendorf tubes containing RNA*later* (Qiagen, Hilden, Germany). Total RNA was extracted using TissueRuptor^®^ and RNeasy^®^ Lipid Tissue Kit (Qiagen). RNA quality was assessed on the Agilent BioAnalyzer 2100 (Agilent Technologies, Santa Clara, USA) and all RNA samples were stored at − 80 °C until their use in hybridization experiments.

### Microarray hybridization

A total of 62 RNA samples were used for gene expression analysis. To determine gene expression profiles, 4x44K oligonucleotide microarrays (Rat Gene Expression 4x44K v3 Microarray Kit, G2519F-028282, Agilent Technologies) were used. The procedures for hybridization using the fluorescent dye Cy3 followed the manufacturer’s protocols (One-Color Microarray-Based Gene Expression Analysis-Quick Amp Labeling and miRNA Complete Labeling and Hyb Kit, Agilent Technologies). A subset of 58 RNA samples was used for miRNA expression analysis, using the whole rat miRNA 8x15K oligonucleotide microarrays (Rat miRNA Microarray slide, G4471A-070154, Agilent Technologies), containing probes for 758 rat miRNAs based on miRBase database (release 21.0). The images were captured by the reader Agilent Bundle according to the parameters recommended for bioarrays and extracted by Agilent Feature Extraction software version 11.5.1.1 (https://www.agilent.com/) for both gene and miRNA expression. Spots with two or more flags (low intensity, saturation, controls, etc.) were considered as NA, that is, without valid expression value. All microarray raw data is deposited in GEO public database (http://www.ncbi.nlm.nih.gov/geo), a MIAME compliant database, under accession reference Series number GSE229760 for mRNA and miRNA data.

### Gene expression analysis

An in-house algorithm in R environment^26^ was used for excluding transcripts presenting one or more missing values (NAs) per group—for the Weighted Gene Co-expression Network Analysis (WGCNA)—or for excluding transcripts presenting more than five NAs per group—for the differential gene expression analysis. It was also used for converting gene expression values to log base 2. Through this procedure we obtained five gene expression matrices: (i) four for the WGCNA—one for each time-interval, and (ii) one for the differential gene expression analysis. The WGCNA data matrices presented 8786 Gene Ontology (GO) annotated genes (for the P12 interval), 7507 genes (for the P30 interval), 4568 genes (for the P60 interval), and 4487 genes (for the P120 interval). The differential gene expression matrix had 3010 GO annotated genes. Boxplot analysis was used for outlier detection. Data normalization for each of the data matrices was performed using limma package^27^ in R environment^26^.

The differential gene expression analyses were conducted for four time-intervals to compare HNS vs. HS groups. Comparative analysis was performed in MeV software (version 4.9.0)^28^ using significance analysis of microarray (SAM) and fold-change value ≥ 2.0.

### Weighted gene co-expression network analysis (WGCNA)

Each time-interval GO annotated genes were used for WGCNA. Networks were constructed using the WGCNA package (version 1.69-81; https://horvath.genetics.ucla.edu/html/CoexpressionNetwork/Rpackages/WGCNA/)^29^ in R environment^26^. Pearson’s correlation coefficient was used for obtaining gene co-expression similarity measures and for the subsequent construction of an adjacency matrix using soft power and topological overlap matrix (TOM). Soft-thresholding process transforms the correlation matrix to mimic the scale-free topology (Supplementary Fig. S1 online). TOM was used to filter weak connections during network construction. Module identification is based on TOM and in average linkage hierarchical clustering. Finally, dynamic cut-tree algorithm was used for dendrogram’s branch selection. The module eigengene (ME) is defined as the first principal component of a given module, which can be considered a representative of the gene expression profiles in a module. Module Membership (MM), also known as eigengene-based connectivity (*k*ME), is defined as the correlation of each gene expression profile with the module eigengene of a given module^29^.

#### Module-trait association

Module-trait association analysis was accomplished using the WGCNA package^29^ in R environment^26^. For each time-interval analysis we considered the HS, HNS, and CT groups as specific traits. Only traits presenting three or more samples each were considered in the module-trait association analysis. Subsequently, we obtained the Gene Significance (GS), i.e., a value for the correlation between the specific trait (groups) and gene expression profiles^29^. The mean GS value for a particular module is considered as a measure of the Module Significance (MS). Modules presenting a correlation value |r| ≥ 0.65 and *p*-value < 0.05 were selected for functional analysis.

#### Node categorization

Highly connected module genes were selected based on their belonging to a given module significantly correlated to a particular trait and presenting MM absolute value. Then, the top 10 genes presenting the highest MM absolute values were considered as hubs, *i.e.,* intramodular highly connected genes, that hold the transcriptional network together and are also associated to specific cellular processes or link different biological processes^30^.

### miRNA expression analysis

The miRNA expression data included only miRNAs that presented raw expression value > 4.0. An in-house algorithm in R environment^26^ was used for converting gene expression values to log base 2. Four miRNA data matrices were obtained, one for each time-interval. Boxplot analysis was used for outlier detection. Data normalization for four data matrices was performed using limma package^31^ in R environment^26^.

The differential miRNA expression analyses were conducted for the three groups across four time-intervals. MeV software version 4.9.0 and ANOVA with *p*-value < 0.01 was used for obtaining the differentially expressed miRNAs for all comparisons.

The abundantly expressed miRNAs for the four time-intervals were selected after analyzing the miRNA expression values distribution through a scatter dot plot (Supplementary Fig. S2 online). Cut-off values closer to the inflection point were adopted, as follows: 5.2, 7.5, 6.5, and 6.7 for P12, P30, P60, and P120 intervals, respectively, in the HS group; 5.0, 6.6, 6.5, and 6.9 for P12, P30, P60, and P120 intervals, respectively, in the HNS group; and 5.2, 7.5, 6.5, 6.7, for P12, P30, P60, and P120 intervals, respectively, in the CT group. Moreover, miRNAs exclusively expressed in one particular group were identified by using the Venn diagram online web-based tool (http://bioinformatics.psb.ugent.be/webtools/Venn/).

### Enrichment analysis

Transcriptional modules often represent biological processes that can be phenotype-specific and the functional enrichment of the genes within a module is widely used for disclosing its biological meaning^32^. Here we performed a functional characterization of each network’s highly connected module genes and the differentially expressed genes (DEGs). Enrichment analysis was accomplished by using the Enrichr online web-based tool (http://amp.pharm.mssm.edu/Enrichr/)^33,34^, according to Gene Ontology Biological Process (GO BP), KEGG (Kyoto Encyclopedia of Genes and Genomes**)** Pathways and MiRTarBase microRNA targets dataset (*Homo sapiens* and *Mus musculus*) to evaluate whether modules contain genes that are overrepresented by specific biological functions and pathways, or happen to be miRNA targets, being further associated with the time-intervals P12, P30, P60, and P120. Output GO BP and KEGG terms presenting *p*-value < 0.005 were considered significantly enriched.

### Statistical analysis

MeV (Multiple Experiment Viewer) software (version 4.9.0) was used for differential gene expression analysis between HNS and HS groups through Significance Analysis of Microarray (SAM) and identifying the differentially expressed genes and miRNAs across the four time-intervals for each group (HNS, HS, and CT) using the ANOVA test (*p* < 0.01).

## Results

### miRNA expression analysis

**Figure 1:**
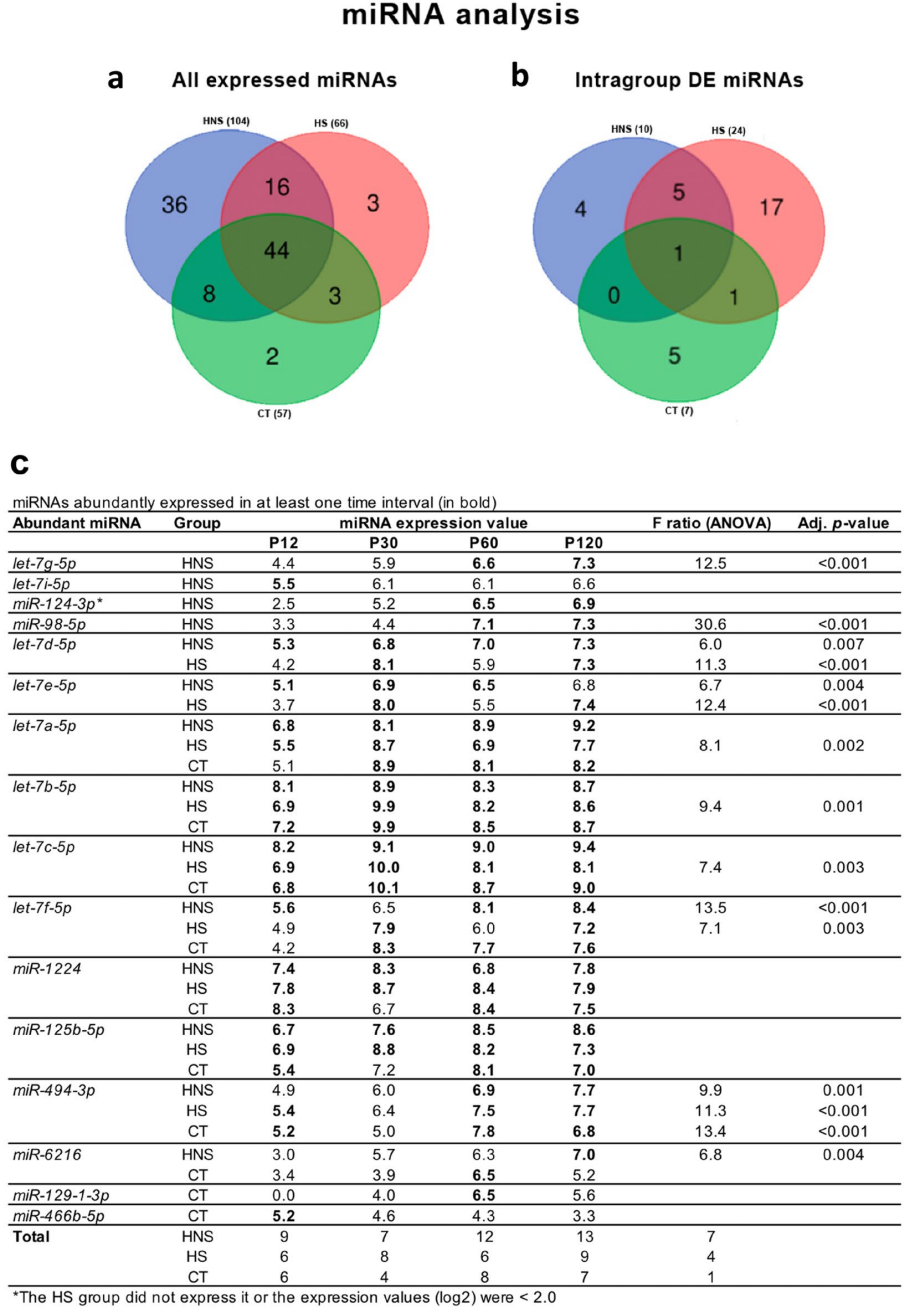
miRNA analysis. (**a**) Venn diagram for all expressed miRNAs identified in the HNS, HS, and CT groups. (**b**) Venn diagram for differentially expressed miRNA in each group across four time-intervals. (**c**) miRNAs abundantly expressed (in bold letters) identified in each group in at least one time-interval.

The three groups—HS, HNS and CT—expressed a total of 104, 66, and 57 miRNAs respectively. The comparative analysis showed that 44 miRNAs were expressed in the three groups, 16 miRNAs were expressed in the groups HNS and HS, eight miRNAs were expressed in the HNS and CT groups, and three miRNAs were expressed in the HS and CT groups (Fig. 1a and Supplementary Table S1 online). It is interesting to note that 36 miRNAs were exclusively expressed in the HNS group, whereas only three were exclusively expressed in the HS group and just two were exclusively expressed in the CT group. However, ANOVA analysis identified more differentially expressed (DE) miRNAs across four time-intervals in the HS group as compared with the HNS and CT groups (Fig. 1b). Moreover, only six out of 24 DE miRNAs were common to the HNS and HS groups.

A total of sixteen abundantly expressed miRNAs were identified (Fig. 1c). Four miRNAs—*let-7g-5p, let-7i-5p, miR-124-3p*, and *miR-98-5p*—were only abundantly expressed in the HNS group. Besides, seven of these miRNAs presented significant expression variation across time-intervals in the HNS group, four in the HS group, and only one in the CT group. It is worth to mention that the HS group did not express *miR-124-3p* in all four time-intervals.

### WGCNA and module-trait correlation analysis

#### P12 time-interval

**Figure 2:**
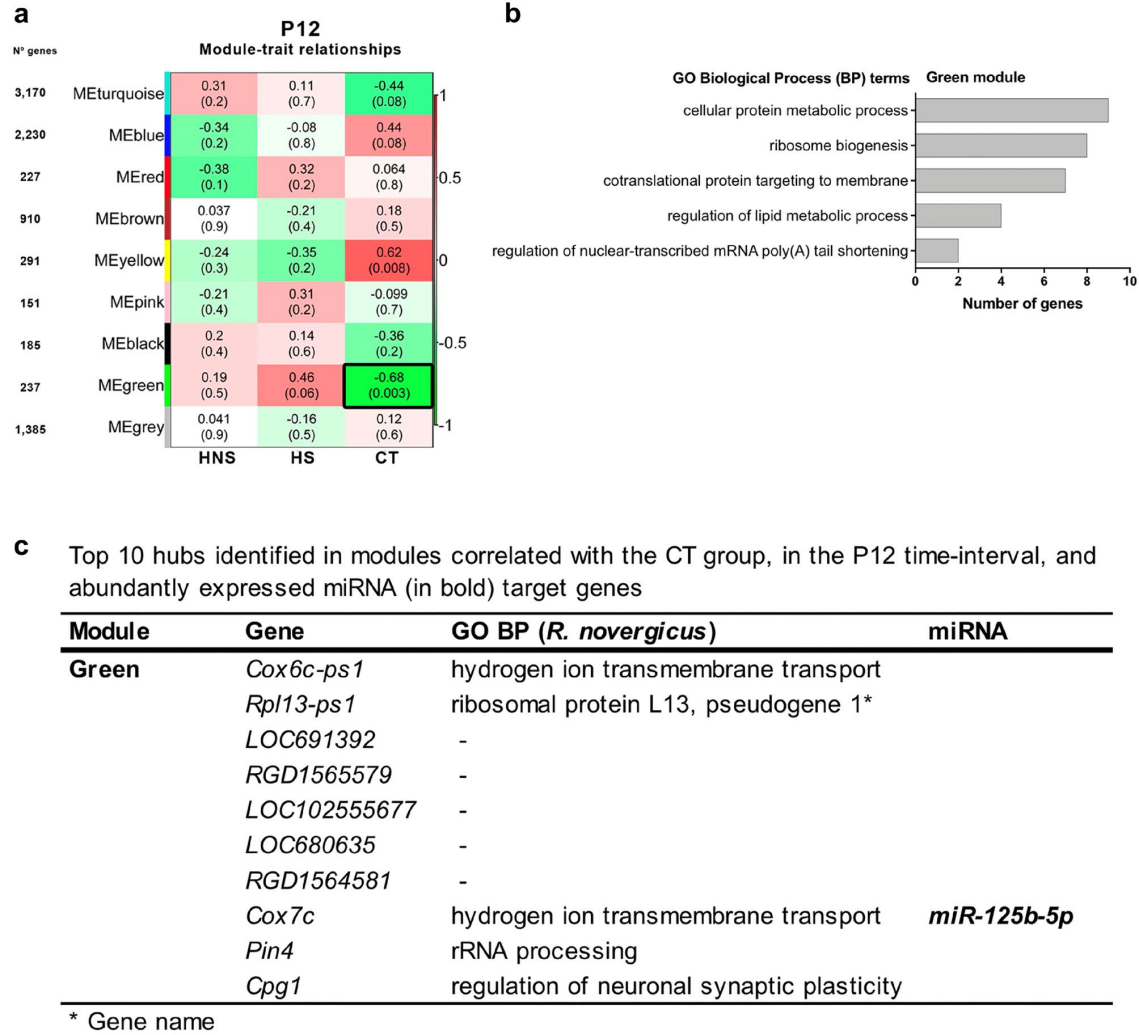
WGCNA analysis for the P12 time-interval. (**a**) Module-trait relationships identified eight modules. Modules’ size ranged from 151 genes in the pink module to 3170 genes in the turquoise module, and each row corresponds to a module eigengene and column to a trait of interest. The numbers stand for the correlation coefficients between the module and a specific trait, with the *p*-values between parentheses. The table is color-coded by correlation according to the color legend. The module highly correlated with the CT group are indicated with black border. (**b**) Histogram of the enriched GO BP terms. (**c**) Hubs of the green module and abundantly expressed miRNA in the CT group and it target gene.

The module-trait correlation analysis identified one module (green module) highly and negatively correlated (r = − 0.68) with the CT group (Fig. 2a). The enrichment analysis for the green module showed that the most represented GO-BP terms are related to cellular processes such as metabolism, transcription, and translation (Fig. 2b, Supplementary Table S2 online). Additionally, three abundantly expressed miRNAs, *let-7b-5p, miR-125-5p,* and *let-7c-5p*, enriched for eight, two, and one genes of this module, respectively (Supplementary Table S3 online). One of these genes, *Cox7c*, is a hub and interacts with *miR-125-5p* (Fig. 2c). Only one hub, *Cpg1*, is involved in regulation of neuronal synaptic plasticity. Cpg1 induction is dependent of the NMDA (N-methyl-d-aspartate) receptor activation and it also encodes a dentate-gyrus-specific protein^35^.

#### P30 time interval

**Figure 3:**
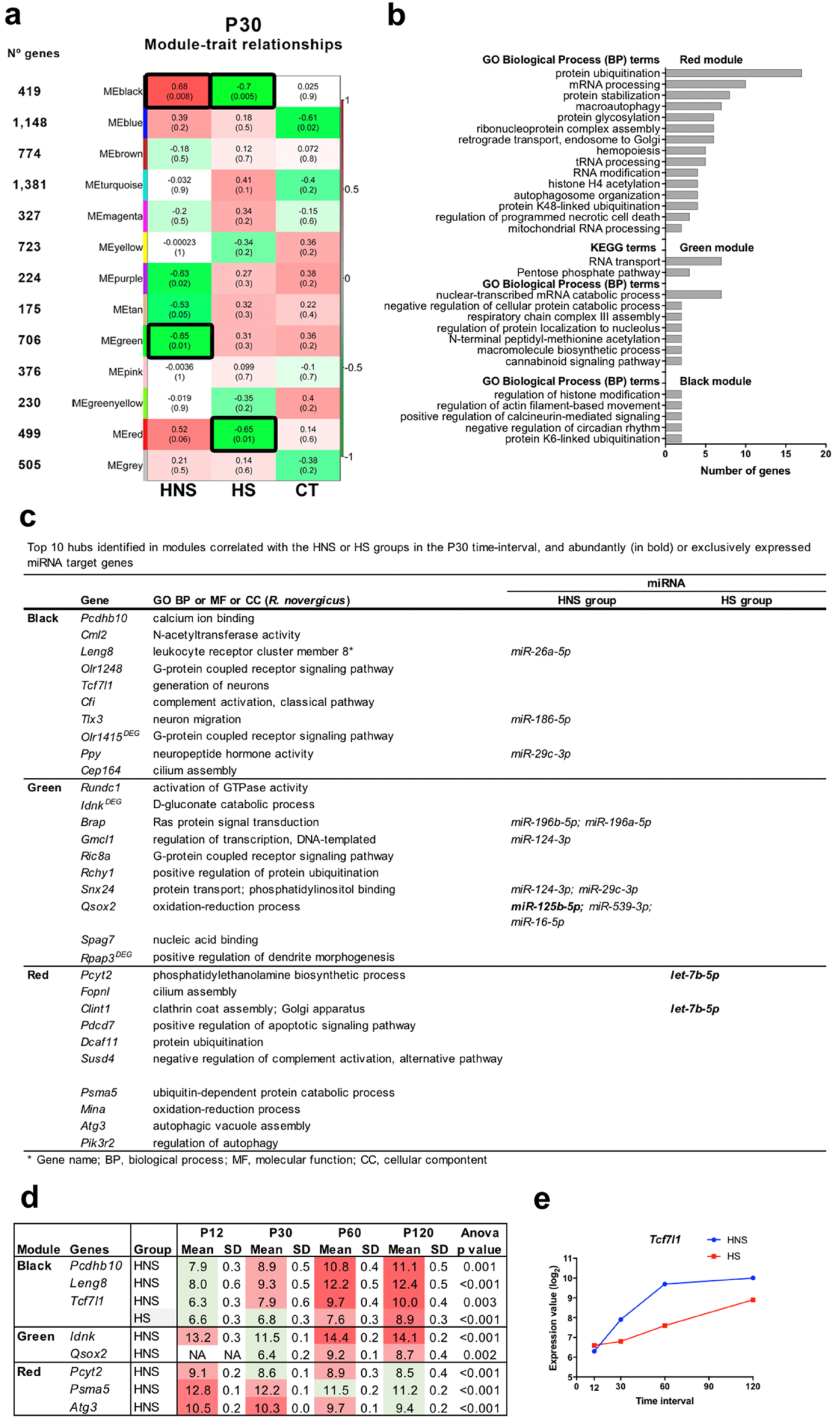
WGCNA analysis for the P30 time-interval. (**a**) Module-trait relationships identified twelve modules. Modules’ size ranged from 175 genes in the tan module to 1,381 genes in the turquoise module. Each row corresponds to a module eigengene and the columns correspond to the traits of interest. The numbers stand for the correlation coefficients between the module and a specific trait, with the *p*-values between parentheses. The table is color-coded by correlation according to the color legend. The modules highly correlated with the HNS or HS groups are indicated with black border. (**b**) Histogram of enriched GO BP and KEGG terms. (**c**) Hubs of this module and miRNA target genes. (**d**) Hubs that presented gene expression variation across the four time-intervals in the HNS and HS groups (p < 0.01 was considered significant). The green color indicates low expression value and the red color intensity is proportional to the expression increase across time intervals. NA stands for gene expression values considered as outliers. (**e**) Scatter plot depicts the expression variation upon *Tcf7l1* in the HNS and HS groups. Each dot represents the mean value in log_2_ scale.

Three modules are highly correlated with at least one group (Fig. 3a). The black module is positively correlated with the HNS group (r = 0.68) and negatively correlated with the HS group (r = − 0.70). The green module is negatively correlated with the HNS group (r = − 0.65). The red module is negatively correlate with the HS group (r = − 0.65).

The black module unveiled that the most represented GO-BP terms are related to ubiquitination and histone modification, circadian rhythm, actin filament-based movement, and calcineurin-mediated signaling (Fig. 3b, Supplementary Table S4 online). Most of the hubs (six out of 10 hubs) are involved in neuronal function (Fig. 3c). One hub is involved in the complement cascade regulation (*Cfi*). It is interesting to note that three hubs—*Pcdhb10, Leng8*, and *Tcf7l1* presented an increase in expression across the four time-intervals in the HNS group (Fig. 3d). In the HS group only *Tcf7l1* presented a similar expression profile to the HNS group, albeit with lower expression values than in the HNS group (Fig. 3e).

The green module revealed that the most represented GO-BP terms are related to transcription, metabolism, and cannabinoid signaling pathway. When considering the KEGG pathways the most represented term is related to RNA transport. It is interesting to note that four hubs interact with six exclusively and one abundantly expressed miRNAs. These genes are involved in oxidative process, signaling, transcription, and protein transport (Fig. 3b,c, Supplementary Table S4 online).

The red module revealed that the most represented GO-BP terms are related to ubiquitination, autophagy, retrograde transport, and cell death. Six of its hubs are involved in immune response (*Susd4*), autophagy (*Atg3, Pik3r2*), apoptosis (*Pdcd7*), and ubiquitination (*Dcaf11, Psma5*) (Fig. 3c, Supplementary Table S4 online). Two hubs, *Pcyt2* and *Clint1*, were enriched for the abundantly expressed *let-7b-5p* in the HS group. *Clint1* codifies for enthoprotin that interacts with clathrin. Altered clathrin-mediated endocytosis contributes to synaptic pathology^36^. Furthermore, all ten hubs in this module did not show variation in gene expression in the HS group. However, in the HNS group, three hubs showed variation in gene expression, with *Psma5* and *Atg3* presenting a decrease in expression over the four time-intervals (Fig. 3d).

Lastly, seven miRNAs were abundantly expressed in the HNS or HS groups and present several genes in the black, green and red modules as targets (Supplementary Table S5 online). Moreover, 21 exclusively expressed miRNAs, including the *miR-124-3p*, in the HNS group present interactions with several genes of the black and green modules, while only two miRNAs exclusively expressed in the HS group enriched for three genes (Supplementary Table S5 online).

#### P60 time-interval

**Figure 4:**
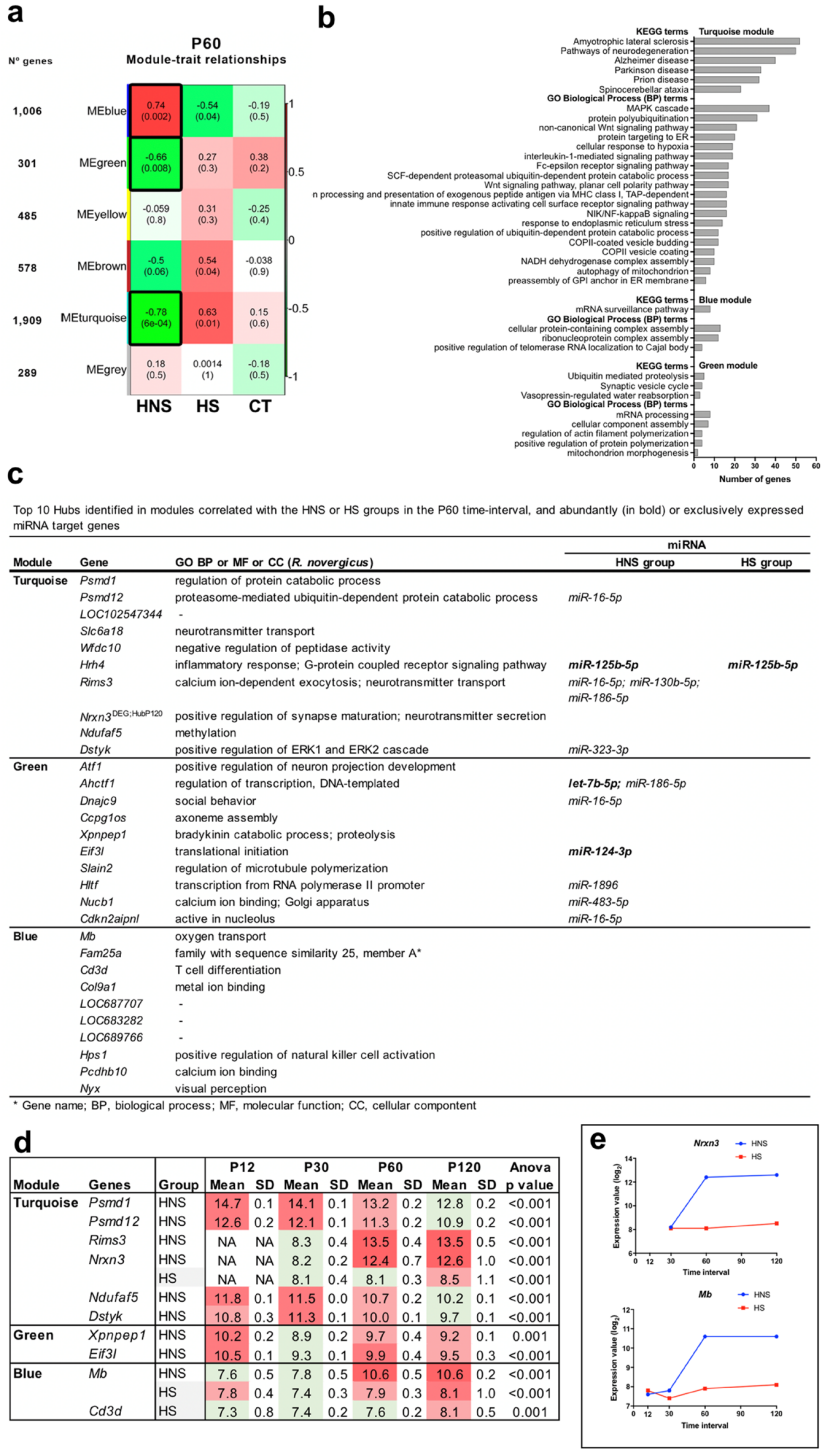
WGCNA analysis for the P60 time-interval. (**a**) Module-trait relationships identified five modules. Modules’ size ranged from 301 genes in the green module to 1,909 genes in the turquoise module. Each row corresponds to a module eigengene and each column to a trait of interest. The numbers stand for the correlation coefficients between the module and a specific trait, with the *p*-values between parentheses. The table is color-coded by correlation according to the color legend. The modules highly correlated with the HNS groups are indicated with black border. (**b**) Histogram of enriched GO BP and KEGG terms. (**c**) Hubs present in highly correlated modules and corresponding miRNA target genes in HS and HNS groups. (**d**) Hubs that presented gene expression variation across the four time-intervals in the HNS and HS groups (p < 0.01 was considered significant). The green color indicates low expression value and the red color intensity is proportional to the expression increase across time intervals. NA stands for gene expression values considered as outliers. (**e**) Scatter plot depicts the expression variation upon *Nrxn3* and *Mb* in the HNS and HS groups. Each dot represents the mean value in log_2_ scale.

Three of the transcriptional modules are highly correlated with the HNS group (Fig. 4a). The blue module is positively correlated (r = 0.74), while the turquoise and green modules are negatively correlated (r = − 0.66 and − 0.78, respectively). The turquoise module showed that the most represented GO-BP terms are related to regulation of immune response and inflammation—highlighting the MAPK cascade and pathways such as interleukin-1-mediated signaling, NIK/NF-kappaB signaling, and Fc-epsilon receptor signaling—with the involvement of the hubs *Psmd1* and *Psmd12,* members of the proteasome 26S subunit, which are also involved in other processes such as polyubiquitination and non-canonical Wnt signaling pathway. Two hubs, *Hrh4* and *Dstyk*, are involved in inflammatory response, being that the Hrh4 was enriched for the abundantly expressed *miR-125b-5p* in the HNS and HS groups while the *Dstyk* was enriched for *miR-323-3p* (exclusively expressed in the HNS group) and it is involved in ERK1/2 cascade. Autophagy and vesicular transport also appeared as enriched terms. Considering the KEGG pathways, the most enriched terms are related to neurodegenerative diseases with the involvement of the same hubs *Psmd1* and *Psmd12* (Fig. 4b,c, Supplementary Table S6 online)*.* Moreover, three hubs (*Slc6a18, Nrxn3,* and *Rims3*) are involved in neuronal activity, with the *Rims3* being enriched for *miR-16-5p*, *miR-130b-5p*, and *miR-186-5p*—all expressed only in the HNS group.

The blue module displayed that the most represented GO-BP and KEGG terms are related to mRNA surveillance pathway, telomerase RNA, and RNA–protein complexes. Two hubs are involved in immune response (*Cd3d, Hps1*).

Four hubs of the turquoise module (*Psmd1, Psmd12, Ndufaf5*, and *Dstyk*) presented a decrease in expression across the four time-intervals in the HNS group (Fig. 4d), while two hubs of this module (*Rims3* and *Nrxn3*) and another hub of the module blue (*Mb*) presented a great increase in expression from P60, as well as other aforementioned hub from the module blue (*Pcdhb10)* at *P30* (Figs. 3d and 4d,e). *Nrxn3*, and *Mb* also presented expression variation in the HS group, but a lower expression in comparison with the HNS group (Fig. 4e).

The green module showed that the most enriched GO-BP terms are related to mRNA processing, cell projection morphogenesis, and protein polymerization—which included the hub *Slain2*. Regarding the KEGG pathways, the most enriched terms are related to ubiquitination and synaptic vesicle cycle*.* It is interesting to mention that two hubs, *Atf1* and *Ccpg1os,* are involved in neuron morphology. Six hubs interact with exclusively or abundantly expressed miRNAs in the HNS group, which four of them are involved in transcription or translation (Fig. 4b,c, Supplementary Table S6 online).

Additionally, all miRNAs abundantly expressed in the HNS and HS groups, except *miR-1224*, interact with several genes of the turquoise, green, and blue modules. It is interesting to note that one of these miRNAs—*miR-124-3p*—not expressed in the HS group, present interaction with 37, 14, and 13 genes of the turquoise, blue, and green modules respectively (Supplementary Table S7 online).

#### P120 time-interval

**Figure 5:**
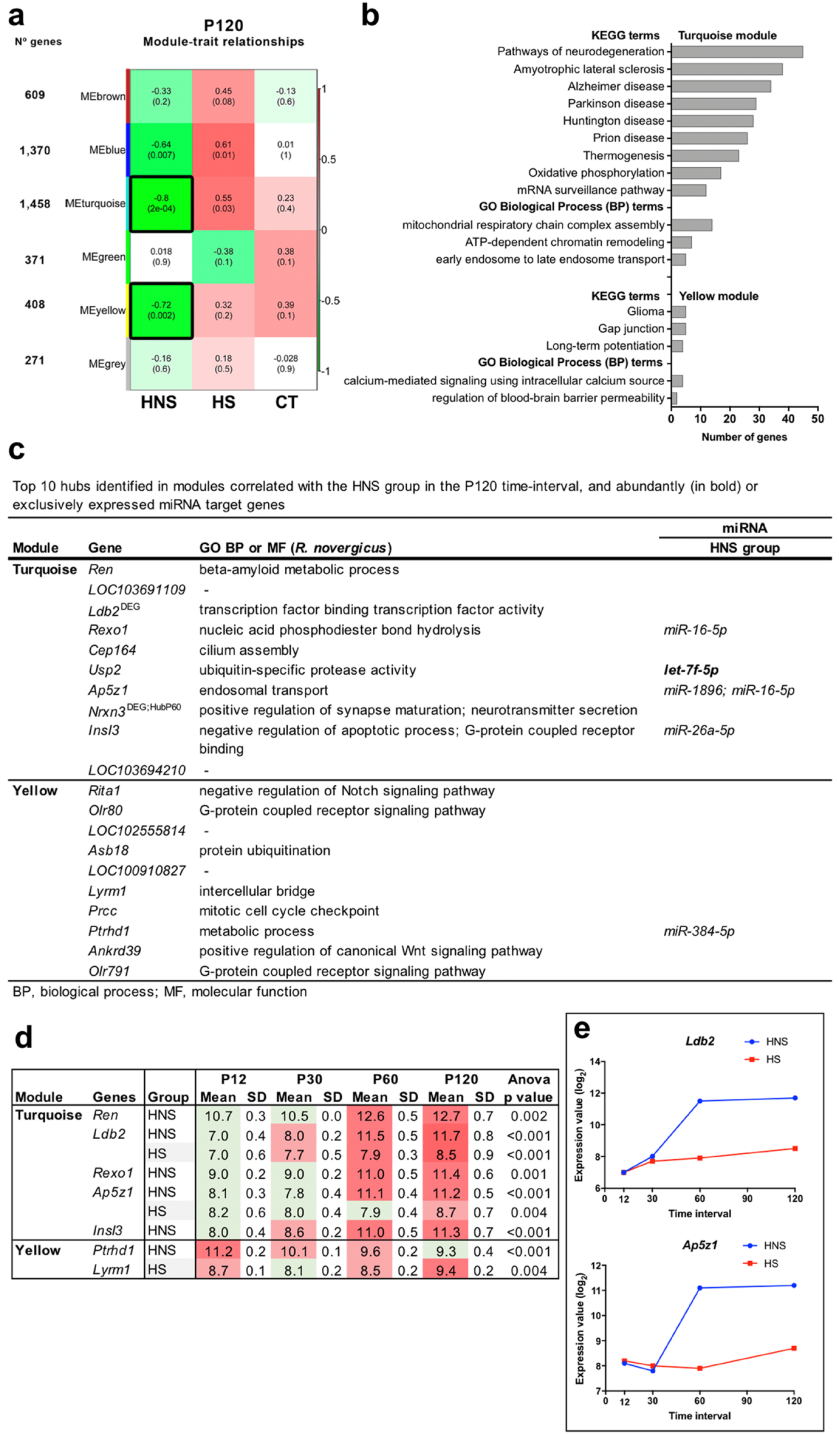
WGCNA analysis for the P120 time-interval. (**a**) Module-trait relationships identified five modules ranging in size from 371 genes in the green modules to 1458 genes in the turquoise module. Each row corresponds to a module eigengene and each column to a trait of interest. The numbers stand for the correlation coefficients between the module and a specific trait, with the *p*-values between parentheses. The table is color-coded by correlation according to the color legend. The modules highly correlated with the HNS groups are indicated with black border. (**b**) Histogram of enriched GO BP and KEGG terms. (**c**) Top ten hubs in the turquoise and yellow modules and their miRNA target genes. (**d**) Hubs that presented gene expression variation across the four time-intervals in the HNS and HS groups (p < 0.01 was considered significant). The green color indicates low expression value and the red color intensity is proportional to the expression increase across time intervals. (**e**) Scatter plot depicts the expression variation upon *Ldb2* and *Ap5z1* in the HNS and HS groups. Each dot represents the mean value in log_2_ scale.

The turquoise and yellow modules are highly and negatively correlated with the HNS group (r = − 0.80 and r = − 0.72, respectively) (Fig. 5a). The turquoise module showed that the most represented GO-BP terms are related to mitochondrial respiratory chain complex, chromatin remodeling, endosome transport, and apoptotic process (Fig. 5b, Supplementary Table S8 online). The hubs *Ap5z1* and *Insl3* are involved in endosomal transport and apoptosis, respectively, and enriched for exclusively expressed miRNAs in the HNS group (Fig. 5c). The most enriched terms in KEGG pathways are neurodegeneration pathways and neurodegenerative diseases, thermogenesis, and oxidative phosphorylation*. Nrxn3* is involved in synapse transmission, being also a hub in the P60 time-interval. Two hubs, *Ldb2* and *Ap5z1*, in addition to the aforementioned *Nrxn3* at P60, showed expression variation across the four time-intervals in both HNS and HS groups (Fig. 5d) with great increase in expression in the HNS group from P60 (Figs. 4e and 5e).

The miRTarBase enrichment analysis showed that 10 out of 13 miRNAs abundantly expressed in the HNS group present interaction with several genes, including the *miR-124-3p*. In addition, 25 miRNAs were expressed only in the HNS group and presented interactions with many genes as well. (Supplementary Table S9 online). Only one hub *Usp2* was enriched with *let-7f-5p*, which is abundantly expressed in the HNS group (Fig. 5c).

The yellow module revealed that the most represented GO-BP terms are related to regulation of blood–brain barrier permeability and calcium-mediated signaling, while the KEGG terms are related to long-term potentiation and gap junction*.* Two hubs, *Olr80* and *Olr791*, are olfactory receptors and are involved in G-protein receptor signaling. The hub *Rita1* is related to the regulation of Notch signaling (Fig. 5c). The miRTarBase enrichment analysis showed that 10 miRNAs abundantly expressed in the HNS group interacted with many genes, including the *miR-124-3p*. In addition, 16 miRNAs expressed only in the HNS group presented interaction with several genes (Supplementary Table S9 online). Only one hub *Ptrhdl* present interaction with *miR-384-5p*, which is expressed only in the HNS group (Fig. 5c).

### Differentially expressed genes analysis for the HNS vs. HS groups

**Figure 6:**
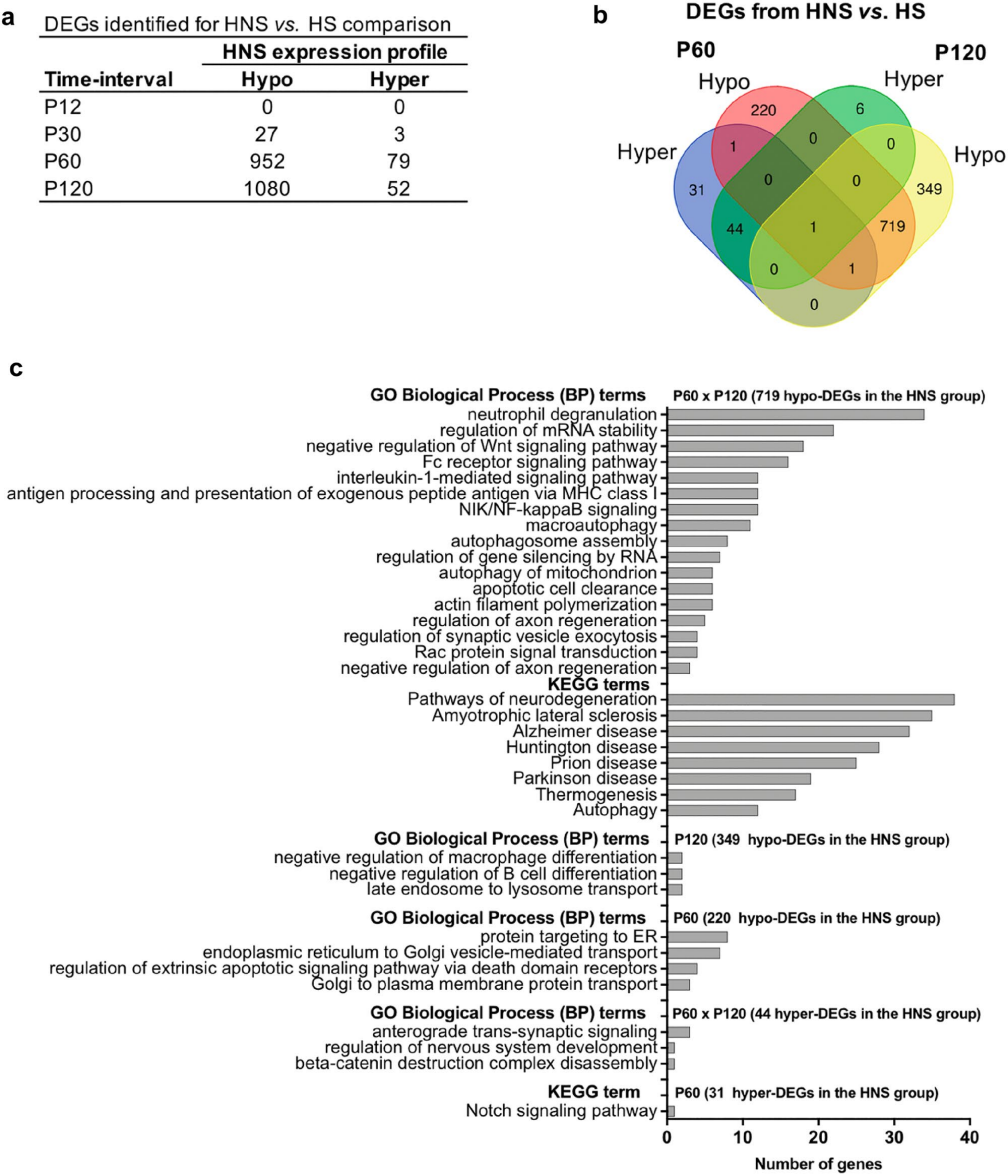
Differentially expressed genes (DEGs) analysis. (**a**) DEGs identified in the HNS vs. HS group pairwise comparisons for four time-intervals. (**b**) Venn diagram for DEGs identified in the P60 and P120 from HNS vs. HS comparisons. (**c**) Enrichment analysis for DEG sets from five Venn diagram sections that appears in (**b**).

Differentially expressed gene (DEG) analysis focused on identifying ventral CA3 gene expression differences between HS and HNS groups in four time-intervals. Noteworthy, no DEG was identified in the P12 interval. However, the other three pairwise comparisons identified an increase in the number of DEGs across P30 to P120 time-intervals (Fig. 6a). Most of the DEGs were hypo-expressed in the HNS group compared with the HS group, i.e., 24 out of 27 DEGs in the P30, 952 out of 1031 DEGs in the P60, and 1080 out of 1132 DEGs in the P120 (Supplementary Tables S10–S12 online).

It is interesting to note that both P60 and P120 time-intervals showed a great number of hypo-expressed genes in the HNS group in comparison with the HS group. Given this similar differential expression profile, we compared these gene sets by Venn diagram and showed that 719 hypo- and 44 hyper-expressed genes were in the overlap area of P60 and P120 (Fig. 6b).

The enrichment analysis of the DEG sets for all comparisons are listed in Supplementary Tables S13, S14 online. In P30, the GO-BP terms such as anion transport and translation were found for hypo-expressed genes in the HNS group compared with the HS group.

The functional analyses for the P60 and P120 time-intervals were performed on the DEG sets obtained in each Venn diagram sections (Fig. 6b). The most enriched terms in KEGG pathways—for the same 719 hypo-expressed genes in HNS group in the P60 and P120—are neurodegeneration pathways and neurodegenerative diseases; while for GO-BP, the most enriched terms are inflammation and immune response such as Fc receptor signaling, interleukin-1-mediated signaling, NIK-NF-kappaB signaling, and neutrophil degranulation, and regulation of axon regeneration. For 220 or 349 hypo-expressed genes in HNS group only in the P60 or P120, respectively, the GO-BP enrichment analysis revealed terms involved in protein target to ER and apoptosis, or mitochondrial translation and transcription. The enriched terms in GO-BP for the same 44 hyper-expressed genes in HNS group in the P60 and P120 are anterograde trans-synaptic signaling, beta-catenin destruction, and nervous system development; and for 31 hyper-expressed genes in HNS group only in the P60, the Notch signaling pathway was found as enriched in KEGG (Fig. 6c, Supplementary Table S12 online).

**Figure 7:**
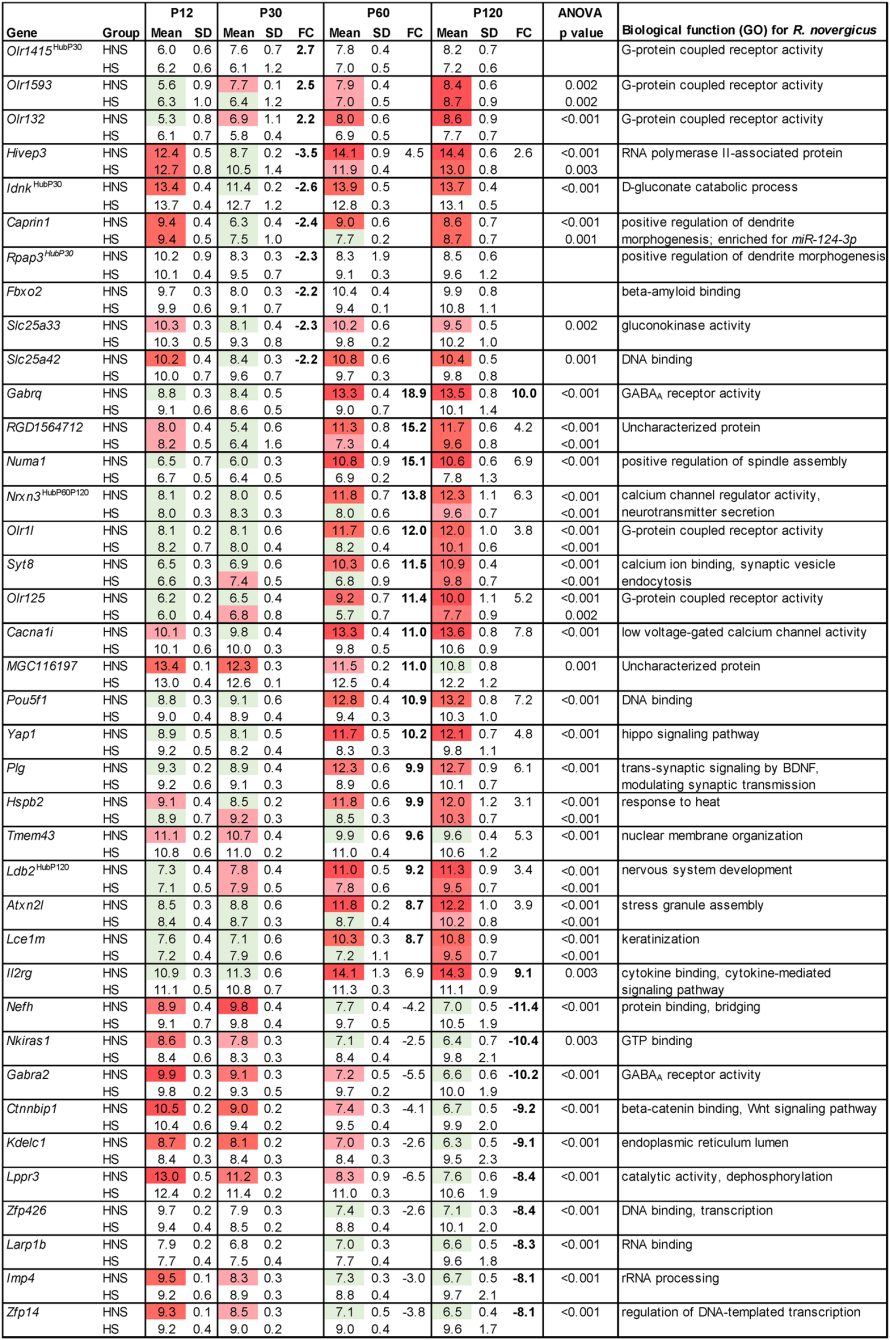
DEGs identified in each pairwise comparison for P30, P60 and P120 time-intervals. Mean expression value in log_2_ scale in all four time-intervals for HNS and HS groups. Fold change (FC) was calculated from gene expression values; positive or negative values indicate hyper- or hypo-expression in the first group of the pairwise comparison (HNS *vs*. HS); values in bold stand for top 10 DEGs in the comparison or FC > 8.0 for P60 and P120 time-intervals. ANOVA test was performed to identify DEGs that presented gene expression variation across the four time-intervals in the HNS and HS groups (p < 0.01 was considered significant). The green color indicates low expression value and the red color intensity is proportional to the expression increase across time intervals.

Additionally, for the P30 time-interval we selected the top 10 DEGs presenting highest fold change values, while for P60 and P120 we adopted an 8.0-fold change cut-off (Fig. 7). In the P30, three DEGs were also hubs in modules of P30 network. One of them, *Olr1415*, was hyper-expressed in the HNS group (HNS vs. HS) and is involved in G-protein coupled receptor activity. This DEG was also a hub in the black module (positively correlated with the HNS group). Other two genes, *Idnk* and *Rpap3*, were hypo-expressed in the HNS group and as a hub in the green module (negatively correlated with the HNS group). Additionally, *Rpap3* and *Caprin1* (hypo-expressed in the HNS group), enriched for *miR-124-3p*—exclusively expressed in the HNS group—are involved in positive regulation of dendrite morphogenesis. Seven of the 10 DEGs showed variation in gene expression over the four time-intervals in the HNS group, of which three of them also showed variation in expression in the HS group. Furthermore, most of these DEGs decreased their expression at P30 and then, at P60, returned to values similar to those seen at P12 or even increased their expression from P60.

In P60, all top DEGs were hyper-expressed in the HNS group in comparison with the HS group. Nine genes—*Gabrq, Nrxn3, Syt8, Cacnali*, *Numa1, Olr11, Orl125, Plg,* and *Ldb2*—are involved in neuronal activity. Two of them—*Nrxn3* and *Ldb2*—are hubs in the transcriptional module related to neurodegenerative diseases, i.e., *Nrxn3* in P60 and P120, and *Ldb2* in P120*. Gabrq*, that codifies for GABA_A_ receptor subunit, was the highest hyper-expressed gene (fold-change, FC = 18.9). *Nrxn3, Syt8,* and *Cacnali* are involved in calcium channel activity. Two DEGs, *Plg* and *Hspb2,* are involved in neuroprotection. All DEGs except *Tmem43* and *MGC116197* showed increased gene expression from P60 in the HNS group. It is noteworthy that in the HS group nine of the 17 DEGs also showed increase in gene expression, but only in P120.

In P120, ten out of 12 DEGs were hypo-expressed in the HNS group compared with the HS group. *Gabrq*, involved in GABAergic activity and *Il2rg* participates in cytokine signaling were hyper-expressed. Another DEG involved in GABAergic activity is *Gabra2*, but it was hypo-expressed in the HNS group. All DEGs presented gene expression variation only in HNS group, of which 10 DEGs showed decreased gene expression and other two, *Gabrq* and *Il2rg*, showed a high increase in expression from P60.

## Discussion

Here we used ventral CA3 transcriptomic analyses for investigating a rat model of hyperthermia-induced febrile seizures^11^ focusing on those animals that did not present seizure after the hyperthermic insult. There is an analogous situation in human epilepsy, where the majority of the infants with a history of febrile seizures does not develop epilepsy^6^. The cellular and molecular mechanisms determining susceptibility or not to febrile-induced epilepsy remain largely unknown. The unveiling of these mechanisms is of paramount importance for developing new therapeutic strategies for prevention or treatment of the progression of MTLE patients with history of febrile seizure during early childhood.

The first question we approached was: do the HS and HNS groups present different ventral CA3 transcriptomic responses after the hyperthermic insult? Comparative analysis between the HNS group and the HS or CT groups revealed that the main transcriptional differences are related to modules involved in inflammatory pathways such as NIK/NF-kβ, ERK1/ERK2, Notch, MAPK, IL-1, and Wnt signaling pathways. Phagocytosis and complement system were also found. Indeed, these modules were negatively correlated with the HNS group. Several studies pointed out neuroinflammation as the main response after febrile seizures, as well as the infiltration of peripheral immune cells such as macrophages, neutrophils, and lymphocytes^6,14,37^. Moreover, it was shown by immunohistochemistry that inflammatory markers can be observed in the hippocampus of rats after hyperthermic seizure and in the surgically resected tissue from patients with drug-resistant focal-onset epilepsy^37,38^. Our results, in accordance with those previous findings, show that in the HS group the neuroinflammatory response was in fact prolonged and may be associated to cellular disfunction and epileptogenesis.

It is also well established that miRNAs play an important role in epileptogenesis^39^. These small molecules are involved in ensuring the robustness and adequacy of gene expression and their dysregulation has been associated to the pathological processes of epilepsy, both in animal models and in human patients^39,40^. Functional studies in animal models indicate that miRNAs participate in repair processes as well as in maladaptive changes^40^, since they are important regulators of cell apoptosis, regenerative process, and immune responses^39^. Here, we found that 36 miRNAs were exclusively expressed in the HNS group while only three and two miRNAs were exclusively expressed in the HS group and CT group, respectively. Moreover, four miRNAs—*let-7 g-5p, let-7i-5p, miR-124-3p*, and *miR-98-5p*—were abundantly expressed only in the HNS group, and *miR-124-3p* was not expressed in the HS group.

In the human brain, as shown by several studies, a given mRNA is commonly either targeted by a specific and abundant miRNA or by multiple and low-abundance miRNAs^40^. Considering this fact, it is interesting to mention the *miR-124-3p*, that is not expressed in HS group or is expressed in a concentration lower than detectable throughout the period under investigation. Furthermore, in the HNS group, from P30 onwards, the expression of this miRNA increases and becomes abundant in the P60 and P120 intervals. The *miR-124-3p* enriched for three hubs (*Gmcl1, Snx24*, and *Eif3l*) of those modules negatively correlated with the HNS group and enriched for inflammatory pathways or neurodegenerative diseases. The expression of miR-124, a brain-specific miRNA, was found suppressed in patients with epilepsy and in rats after drug-induced seizures^41^. Evidence obtained in rats showed that miR-124 attenuates epileptogenesis via NRSF (neuron restrictive silencer factor) while promoting epilepsy via inflammation^42^.

Our next question focused on when transcriptional changes occur along the different ages after hyperthermic induction that correlated to different phases of the disease in human^12,13^, because the timing of therapy intervention is critical to become effective or even avoid epileptogenesis^37^. For example, inflammatory processes are involved in tissue repair and homeostasis maintenance after brain injury^37^. However, studies have shown that brain inflammation promotes neuronal hyper-excitability causing seizures and driving to a vicious circle^43^. Therefore, therapeutic intervention in neuroinflammatory pathways can be beneficial but also ineffective, or even harmful^37,43^. For that reason, we investigated the transcriptional changes in four different time intervals and performed a comparative analysis between the groups HNS, HS and CT.

The temporal analysis showed that in the P12 interval all animals (i.e., the HNS and HS groups) exposed to the hyperthermic insult responded similarly, as the pairwise comparison did not show a differential transcriptional profile and the WGCNA did not identify transcriptional modules correlated to these groups. However, a previous study conducted in our laboratory showed that some biological functions related to inflammation, apoptosis, and neurogenesis were activated in the ventral CA3 of neonate rats that developed hyperthermic seizures^14^. The present results indicate that pups without seizures also activate those processes, but somehow fine-tuned them to do not convulse. It is interesting to mention that the miRNA analysis showed transcriptional profile differences between HNS and HS group in this interval. Four miRNAs—*let-7i-5p, let-7d-5p, let-7e-5p*, and *let-7f-5p*—were abundantly expressed only in the HNS group (Fig. 1c).

From P30 time-interval, the animals of the HNS and HS groups began to show an increasing number of differentially expressed genes, where the HNS group showed a hypo-expression profile compared to the HS group. Furthermore, from the P60 and P120 intervals, the transcriptional difference between the two groups seems to “stabilize”. These results may be related to the functional adaptation to the “new normal” in the HNS group and the establishment of the disease in the HS group.

In the P30 interval, the hubs in the highly correlated modules and hyper-expressed DEGs in the HNS group are linked to neurogenesis and important neuronal functions as synapse and neuronal excitability. In addition, six hubs in this module are involved in neuronal function. The hub *Tcf7l1* is involved in neurogenesis and it is linked with Wnt signaling pathway and Alzheimer`s disease^44^. Additionally, *Tcf7l1*, in HNS and HS groups, showed a progressive increase in gene expression across the four time-intervals, but in the HS group it presented a lower expression in comparison with the HNS group. Another hub, *Tlx3,* enriched for the exclusively expressed *mir-186-5p* in the HNS group, is involved in neuronal migration. Moreover, in chicken and mouse, Tlx3 acts as a switch in determining glutamatergic and GABAergic phenotypes^45^. Three hubs, *Olr1248*, *Olr1415*, and *Ppy*, are involved in the G-protein signaling pathway, two of them being olfactory receptors. The expression of olfactory receptors (ORs) is not restricted to olfactory receptor neurons, and it can be found in hippocampal neurons^46^. These proteins belong to G-protein coupled receptor (GPCR) family, which are well known to be involved in synapse and associated with febrile seizures and complex epilepsy^8,47,48^. The hub *Ppy* codifies a neuropeptide and its expression have been found increased in epilepsy animal models^49^. This neuropeptide is already being investigated for use in nanotherapy for the treatment of epilepsy^50^. Moreover, *Ppy* was enriched for *mir-29c-3p*, which was exclusively expressed in the HNS group. The sixth hub *Pcdhb10* encodes a calcium-dependent cell–cell adhesion protein of the cadherin superfamily. PCDHs have been reported to be relevant to epilepsy and neurodegenerative diseases^51^. Defective function of cadherin, such as PCDH19, is known to be related to febrile convulsions and most often patients are drug-resistant to treatment^52^. It is interesting to mention that only in the HNS group *Pcdhb10* presented gradual increase in gene expression across the four time-intervals (Fig. 3d).

The hub and DEG *Rpap3* and the DEG *Caprin1* were hypo-expressed in HNS group and are involved in dendritogenesis. *Rpap3* enriched for *mir-124-3p*, which is exclusively expressed in HNS group. The analysis of the hippocampus of the febrile seizure animal models showed that seizure accelerates neurogenesis and increase dendritic complexity in dentate gyrus contributing, consequently, to neuronal hyperexcitability^53^. Another interesting DEG is the *Fbxo2*, which encodes an amyloid precursor protein (APP) processing regulator. APP is present at synapses and is thought to play a role in the formation and plasticity of neuronal structures^54^. The hub *Cfi*, an inhibitor of the complement cascade activation, that is also identified as a potential therapeutic target for epilepsy^55^. It is known that the persistence of complement activation might contribute to neuroinflammation maintenance^37,43,56^. Moreover, the module enriched for cannabinoid signaling pathway. Endocannabinoid signaling controls excessive neuronal excitability and epileptic seizure^57^. Cannabidiol is extensively investigated for use in the treatment of refractory epilepsy^57^.

The modules highly correlated with HS group as well as its hubs are related to immune response (*Susd4*), autophagy (*Atg3, Pik3r2*), apoptosis (*Pdcd7*), and ubiquitination (*Dcaf11, Psma5*). It is known that dysfunctional autophagy occurs in epilepsy, mainly caused by an imbalance between excitation and inhibition in the brain^58^. A human pathological study of resected temporal lobe cortex showed that the PIK3R2 expression was significantly higher in patients with refractory temporal lobe epilepsy than in those with non-epileptic disorders^59^. SUSD4 is a regulator of the complement system by binding the C1Q domain, but this domain is also a synaptic regulator involved in degradation of the activity-dependent AMPA receptor subunit GluA2^60^.

In the P60 and P120 intervals, the modules negatively correlated with HNS group and their hubs are involved in pathways of neurodegenerations. The hub *Dstyk,* involved in ERK1/ERK2 cascade, interacts with miR-323-3p (exclusively expressed in the HNS group). ERK1/2 cascade that is an effector of neuronal death and neuroinflammation in many neurodegenerative diseases^61^. Other hub is *Rita1*, which is related to the regulation of Notch signaling. Interestingly, in a rat model of temporal lobe epilepsy, expression of Notch signaling increased after status epilepticus in hippocampus tissue^62^.

It is important to highlight 11 genes involved in neuronal activity. The hub *Rims3,* hyper-expressed in HNS group from P60, encodes a pre-synaptic protein and is involved in synaptic vesicle cycling^63^ and enriched for three exclusively expressed miRNAs—*miR-16-5p*, *miR-130b-5p*, and *miR-186-5p*—in the HNS group. The hub *Slc6a18,* a neurotransmitter, was found hypo-expressed in rats submitted to hyperthermic insult and developed seizures when compared with rats not submitted to hyperthermic conditions, as reported in our previous work^14^. Nine DEGs—*Gabrq, Nrxn3, Syt8, Cacnali*, *Numa1, Olr11, Orl125, Plg,* and *Ldb2*—of which two of them, *Nrxn3* (also hub at P60 and P120) and *Ldb2* (also hub at P120). *Nrxn3* encodes for an α-neurexin isoform which is a neurotransmitter expressed in GABAergic interneuron involved in pre-synaptic modulation of the excitatory synapses^64,65^ and is highly expressed in many GABAergic interneuron subtypes in hippocampus^65^. The highest hyper-expressed gene in the HNS group was *Gabrq*, a GABA_A_ receptor involved in the modulation of inhibitory synapses^66^. Another DEG involved in GABAergic activity is *Gabra2*, which was hypo-expressed in the HNS group at P120. It is well recognized that GABA receptors are commonly associated with epilepsy^67^. Additionally, many studies suggest that deficits in the GABA_A_ receptors such as GABRA2 and GABRA5 contribute to central nervous system disorders such as anxiety disorders, epilepsy, schizophrenia, and insomnia^68,69^. *Syt8* and *Cacnali,* are involved in calcium channel activity. The calcium channelopathies are associated with several neurological disorders including epilepsy, migraine, and ataxia^70^. Variants in *CACNA1I* are related to epilepsy^70^ and to neurodevelopmental disorders, with a phenotypic spectrum ranging from borderline intellectual functioning to a severe neurodevelopmental disorder with epilepsy^71^. *Syt8* encoded protein exists as a membrane bound form in primary neurons^72,73^.

It is well known that dysregulation in the neurotransmitter expression is associated with epilepsy and other neurological disorders. Moreover, in the P60 interval many highly hyper-expressed DEGs in the HNS group compared with the HS group are involved in neuronal activity, possibly related to neuronal loss in the HS group. A previous study conducted by our group in hippocampal tissues obtained from refractory temporal lobe epilepsy patients with early (infant) or late (adult) disease onset showed a decreased expression of genes involved in neuronal activity in CA3 in those hippocampi with neuronal loss and in patients presented memory and executive function impairment^74^. Furthermore, studies in rat model of febrile seizure have shown a cognitive and memory impairment in affected adult animals^75^.

Two DEGs deserve mention, *Plg* and *Hspb2,* which are hyper-expressed in the HNS group from P60 and are involved in neuroprotection. *Plg* codifies for plasminogen and the plasminogen activator (PA) system promotes neurorepair in the ischemic brain^76^. PA system components influence neuronal activity, inflammatory response, and contribute to remodeling of the neuronal networks occurring in epileptogenic lesions^77^. *Hspb2*, encodes a heat shock protein (Hsp) and express only in hippocampus and cortex. The increased expression of Hsps induced by heat shock and their constant neuronal expression may be involved in neuroprotection^78^. One gene *Yap1* involved in hippo signaling and in neurogenesis and astrogenesis^79^.

Moreover, the temporal analysis of the gene expression along the four intervals showed that the hubs and DEGs mentioned above vary similarly between the two groups, however the HS group presented a lower expression than the HNS group. These results indicate that the hyperthermia-insulted pups who did not have ensuing seizures (HNS) can modulate the inflammatory response after the insult, presenting better tissue adaptation and repair than the HS group.

This study has some limitations. One is related to use an animal model of hyperthermic seizure in pups to mimic febrile seizure in early childhood, but for now it is the well stablished animal model to investigate the hippocampal changes induced by prolonged hyperthermic seizure. The other limitation was the inclusion of only male rats in the study. This approach was adopted for feasibility reasons, because a much larger number of animals would be needed to analyze sex-paired samples at all four time-intervals, to avoid possible transcriptome confounding effects due to variable estrogen levels on neuronal excitability.

In conclusion, this study revealed temporal transcriptional differences between rats that developed (HS group) or did not develop seizures (HNS group) after prolonged hyperthermic insult. Our results indicate that in the HS group the neuroinflammatory response was prolonged and may be associated with cellular dysfunction and epileptogenesis. Transcriptional modules are involved in inflammatory pathways such as NIK/NF-kβ, ERK1/ERK2, Notch, MAPK, IL-1 and Wnt signaling pathways, as well as many genes—*Tcf7l1, Tlx3, Rpap3, App, Yap1, Ldb2*, and *Hspb2*—involved in neurogenesis, dendritogenesis and neuroprotection were differentially expressed in the HNS and HS groups. Furthermore, we identified a miRNA—*miR-124-3p*—expressed exclusively and abundantly in the HNS group. Finally, this approach allowed the identification of potential therapeutic targets, such as pathways, genes and miRNA, for the treatment of MTLE patients with a history of febrile seizures in early childhood.

## Supplementary Information

Supplementary Information.

## Data Availability

The datasets generated during and/or analyzed during the current study are available from the corresponding author on reasonable request. All microarray raw data have been deposited in GEO public database (http://www.ncbi.nlm.nih.gov/geo), a MIAME compliant database, under reference Series accession number GSE229760 for mRNA and miRNA data, token gnmhoamcbvithml. All data will be released with paper publication.
