# Supplementary material for: Transcriptomic analysis reveals distinct adaptive molecular mechanism in the hippocampal CA3 from rats susceptible or not-susceptible to hyperthermia-induced seizures
Source: Sci Rep. 2023 Jun 24;13:10265. doi: 10.1038/s41598-023-37535-w (PMC10290664; doi:10.1038/s41598-023-37535-w)
Supplement: Supplementary file 1 — Supplementary Information. [file 41598_2023_37535_MOESM1_ESM.zip › SupplementaryInformation/Supplementary Information_BandoSY et al.docx]

*Supplementary Information*

**Transcriptomic analysis reveals distinct adaptive molecular mechanism in the hippocampal CA3 from rats susceptible or not-susceptible to hyperthermia-induced seizures**

Silvia Y. Bando^1*^, Fernanda B. Bertonha^1^, Pedro H. N. Menezes^1^, Nathália A. Khaled^1^, Paula Santos^1^, Mara de S. Junqueira^2^, André K. Takahara^1^, Roberto M Cesar Jr^3^, Carlos A. Moreira-Filho^1^

^1^ Department of Pediatrics, Faculdade de Medicina da Universidade de São Paulo, São Paulo, SP, 05403-900, Brazil.

^2^ Department of Radiology and Oncology, Centro de Investigação Translacional em Oncologia – Instituto do Câncer do Estado de São Paulo, Faculdade de Medicina da Universidade de São Paulo, São Paulo, SP, 05403-000, Brazil.

^3^ Department of Computer Science, Instituto de Matemática e Estatística da Universidade de São Paulo, SP, 05508-040, Brazil.

Silvia Y. Bando, [silvia.bando@fm.usp.br](mailto:silvia.bando@fm.usp.br), <https://orcid.org/0000-0003-3794-2440>

Fernanda B. Bertonha, [fernanda.bernardi@fm.usp.br](mailto:fernanda.bernardi@fm.usp.br), <https://orcid.org/0000-0002-3675-1362>

Pedro H. N. Menezes, [pedronmenezes45@gmail.com](mailto:pedronmenezes45@gmail.com)

Nathália A. Khaled, [nathalia.khaled@gmail.com](mailto:nathalia.khaled@gmail.com)

Paula Santos, [paula.gaader@gmail.com](mailto:paula.gaader@gmail.com), <https://orcid.og/0000-0002-4144-9010>

Mara de S. Junqueira, [mara.junqueira@hc.fm.usp.br](mailto:mara.junqueira@hc.fm.usp.br), <https://orcid.org/0000-0002-2404-6543>

André K. Takahara, [akeiji.taka@gmail.com](mailto:akeiji.taka@gmail.com)

Roberto M. Cesar Jr, [rmcesar@usp.br](mailto:rmcesar@usp.br), <https://orcid.org/0000-0003-2701-4288>

Carlos A. Moreira-Filho, [cmoreira@usp.br](mailto:cmoreira@usp.br) , <https://orcid.org/0000-0003-3433-4714>

^*^ Correspondence: Silvia Yumi Bando, PhD; [silvia.bando@fm.usp.br](mailto:silvia.bando@fm.usp.br); tel 55-11-3061-8449

**Supplementary Figures**

**
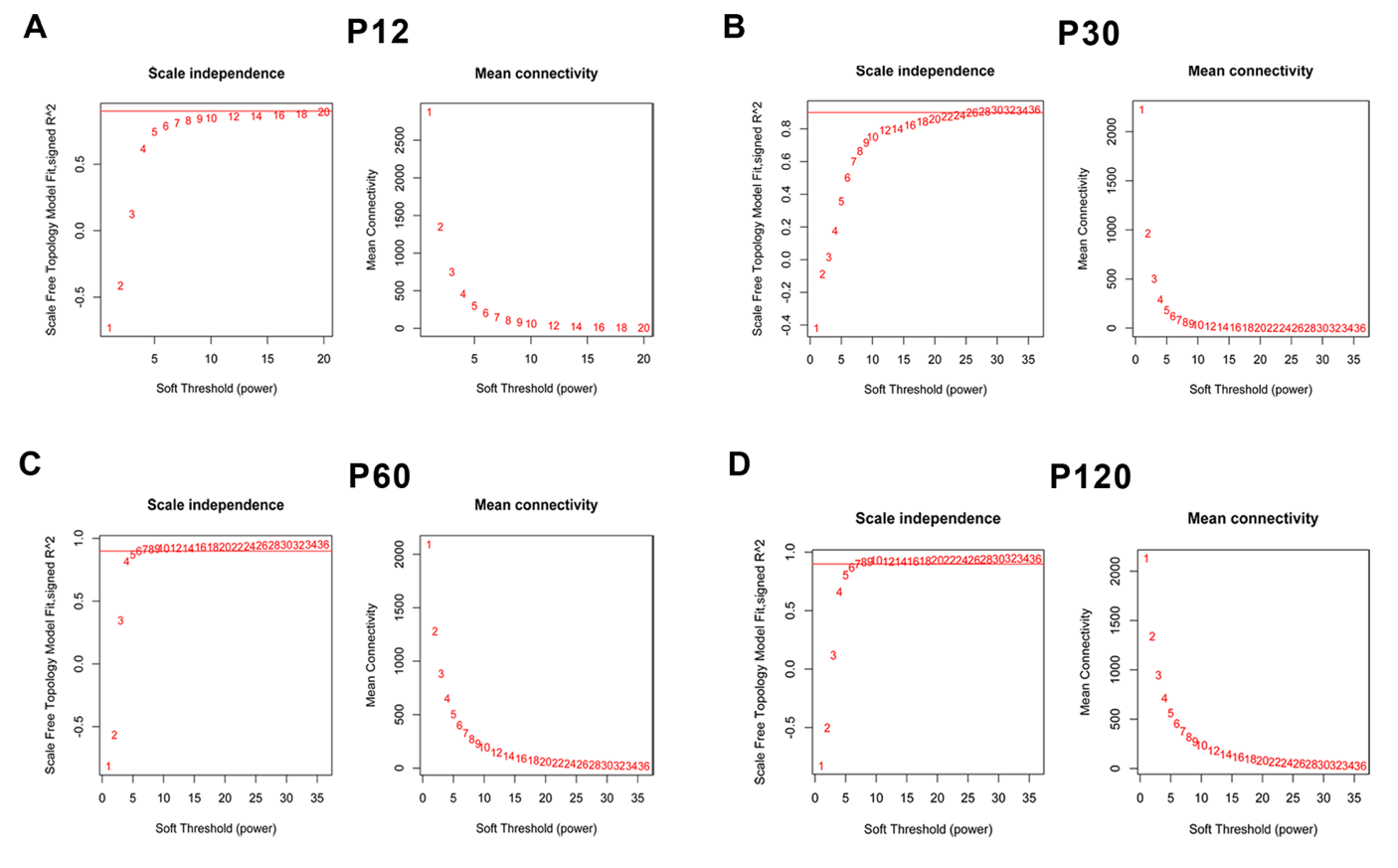
**

**Figure S1.** **WGCNA analysis for each time-interval.** Analysis of P12 network topology for various soft-thresholding powers (**A**). Analysis of P30 network topology for various soft-thresholding powers (**B**). Analysis of P60 network topology for various soft-thresholding powers (**C**). Analysis of P120 network topology for various soft-thresholding powers (**D**). For each time-interval, the left panel shows the scale-free fit index (y-axis) as a function of the soft-thresholding power (x-axis), while the right panel displays the mean connectivity (degree, y-axis) as a function of the soft-thresholding power (x-axis).


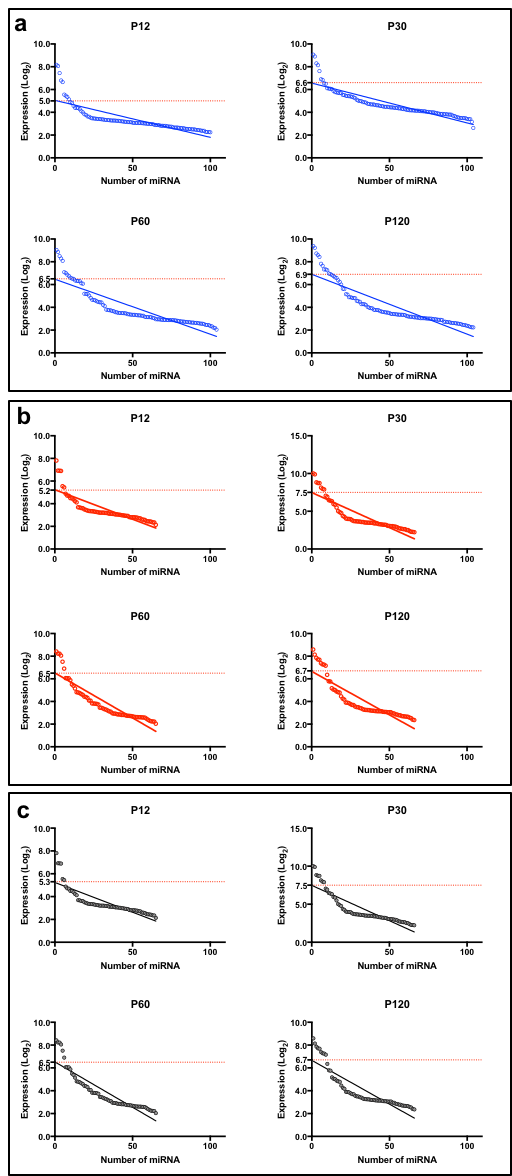


**Figure S2. Abundantly expressed miRNA identification.** Scatter plot of the miRNA expression values in log_2_ scale obtained in the P12, P30, P60, and P120 time-intervals for groups HNS (**a**), HS (**b**), and CT (**c**). Cut-off value is indicated by red dot line.

**Supplementary Tables**

**Table S1.** miRNAs expressed in each group in at least one time-interval. Expression values in log_2_ scale. The expression values in bold letters are differentially expressed miRNA across four-time intervals identified in each group. ND, miRNA expression not detected by microarray analysis

**Table S2.** Enrichment analysis for the modules correlated with the CT group in the P12 time-interval. Terms in bold appear in the histogram

**Table S3.** miRNAs and target genes identified in the highly correlated module with the CT group in P12 time-interval

**Table S4.** Enrichment analyses for the modules correlated with the HNS and HS groups in the P30 time-interval.Terms in bold appear in the histogram

**Table S5.** miRNAs and target genes identified in the highly correlated modules with the HNS or HS groups in P30 time-interval

**Table S6.** Enrichment analyses for the modules correlated with the HNS group in the P60 time-interval.Terms in bold appear in the histogram

**Table S7.** miRNAs and target genes identified in the highly correlated modules with the HNS group in P60 time-interval

**Table S8.** Enrichment analyses for the modules correlated with the HNS group in the P120 time-interval.Terms in bold appear in the histogram

**Table S9.** miRNAs and target genes dentified in the highly correlated modules with the HNS group in P120 time-interval

**Table S10.** DEGs identified in the P30 time-interval for HNS *vs*. HS comparison. Average expression value in log_2_ scale. Fold change was calculated from gene expression value, positive or negative values indicate hyper- or hypo-expressed in the first group in the pairwise comparison

**Table S11.** DEGs identified in the P60 time-interval for HNS *vs*. HS comparison. Average expression value in log_2_ scale. Fold change was calculated from gene expression value, positive or negative values indicate hyper- or hypo-expressed in the first group in the pairwise comparison

**Table S12.** DEGs identified in the P120 time-interval for HNS *vs*. HS comparison. Average expression value in log_2_ scale. Fold change was calculated from gene expression value, positive or negative values indicate hyper- or hypo-expressed in the first group in the pairwise comparison

**Table S13**. Enrichment analyses for the DEGs found in the P30 time-interval from comparison between the HNS *vs*. HS groups

**Table S14**. Enrichment analyses for the DEGs found in the comparison between the HNS *vs*. HS groups, in the P60 and P120 time-intervals. The DEG sets correspond to Venn diagram sections. Terms in bold appear in the histogram
